# KLF4 transcription factor in tumorigenesis

**DOI:** 10.1038/s41420-023-01416-y

**Published:** 2023-04-08

**Authors:** Zhihong He, Jie He, Keping Xie

**Affiliations:** 1 Center for Pancreatic Cancer Research, The South China University of Technology School of Medicine, Guangzhou, China; 2 The South China University of Technology Comprehensive Cancer Center, Guangdong, China; 3 The Second Affiliated Hospital and Guangzhou First People’s Hospital, South China University of Technology School of Medicine, Guangdong, China

**Keywords:** Oncogenesis, Pancreatic cancer

## Abstract

Krüppel-like transcriptional factor is important in maintaining cellular functions. Deletion of Krüppel-like transcriptional factor usually causes abnormal embryonic development and even embryonic death. KLF4 is a prominent member of this family, and embryonic deletion of KLF4 leads to alterations in skin permeability and postnatal death. In addition to its important role in embryo development, it also plays a critical role in inflammation and malignancy. It has been investigated that KLF4 has a regulatory role in a variety of cancers, including lung, breast, prostate, colorectal, pancreatic, hepatocellular, ovarian, esophageal, bladder and brain cancer. However, the role of KLF4 in tumorigenesis is complex, which may link to its unique structure with both transcriptional activation and transcriptional repression domains, and to the regulation of its upstream and downstream signaling molecules. In this review, we will summarize the structural and functional aspects of KLF4, with a focus on KLF4 as a clinical biomarker and therapeutic target in different types of tumors.

## Facts

1. Transcription factors regulate the expression of numerous genes in the organism and play a crucial role in embryonic development, physiological and pathologic processes. The transcription factor Krüppel-like factor 4 is important in the differentiation of epithelial cells and deletion of KLF4 leads to impaired barrier function of skin and death of the newborn.
2. KLF4 is one of the most reported transcription factors among KLF family members, and nearly half of the reports are associated with tumors. Evidently, KLF4 plays an important role in the vast majority of tumors, including lung, breast, prostate, colorectal, pancreatic, liver, ovarian, esophageal, bladder, and brain cancers.
3. Studies over the past three decades have shown that KLF4 inhibits tumor progression in most types of tumors. Therefore, it is considered as an “tumor suppressor”. However, in a few types of tumors, such as breast cancer and glioblastoma, KLF4 appears to be a “oncogene”. Also, there is increasing evidence that KLF4 can promote the precancerous lesions, such as the early lesions of pancreatic cancer, ADM and PanIN.
4. The exact molecular mechanisms how KLF4 executes its function in tumors remains unclear, although the most important molecules downstream KLF4 include P21, P27, P53 and Cyclin-D. Whether KLF4 promotes precancerous lesions in other types of tumors has not been reported.

## Questions

1. Does KLF4, which promotes precancerous lesions of pancreatic cancer, similarly promote the precancerous process in other types of tumors?
2. KLF4 inhibits tumor growth in the advanced stage of pancreatic cancer, while it plays the opposite role in the precancerous lesions. Is this a function of KLF4 itself, involving in completely opposite regulatory mechanisms?
3. Can KLF4 be used as a marker for specific types of tumors or precancerous lesions and be used for early diagnosis and detection through exosomes and other modalities?
4. Can KLF4 be used as a drug target to inhibit tumorigenesis and progression by targeting the transcriptional activation and repression domains of KLF4 with small molecule inhibitors, or altering its transcription and translation?

Krüppel-like Factor 4 (KLF4) is a member of evolutionarily conserved family of zinc finger transcription factors, which was first discovered in the differentiated epithelium, colon and small intestine of newborn mice by Garrett-Sinha and Shields in 1996 [1, 2]. KLF4 is also named as epithelial zinc finger protein (EZF) and gut-enriched Krüppel-like factor (GKLF). Early studies have suggested that KLF4 is expressed in cells with stagnant growth and plays an important role in cell proliferation and differentiation [2]. Later, human KLF4 is cloned and found to locate in chromosome 9q31 [3]. The corresponding protein consists of 513 amino acids, and contains both transcriptional activation and suppression domains, which bind specifically to a responsive element containing the CCACC core sequence [4].

KLF4 is a transcription factor regulating not only cellular physiological processes such as proliferation, differentiation and apoptosis, but also pathogenesis of inflammation and tumorigenesis. However, the mechanism of its numerous physiological and pathological functions remain unclear [5], and the functions of KLF4 in most types of tumors are controversial and confusing [6, 7]. KLF4 maintains a certain cellular status mainly by regulating cell proliferation, differentiation and cycle, e.g., intestinal goblet cells [8]. However, whether these mechanisms are applicable in various types of tumors is unclear. This review summarizes the major functions and regulatory mechanisms of KLF4 in ten common cancers in an attempt to discover its unique tumor regulatory role, and propose KLF4 as a potential therapeutic target and clinical biomarker in tumors, especially pancreatic cancer (PDA).

## Structure and functions of KLF4

**Fig. 1:**
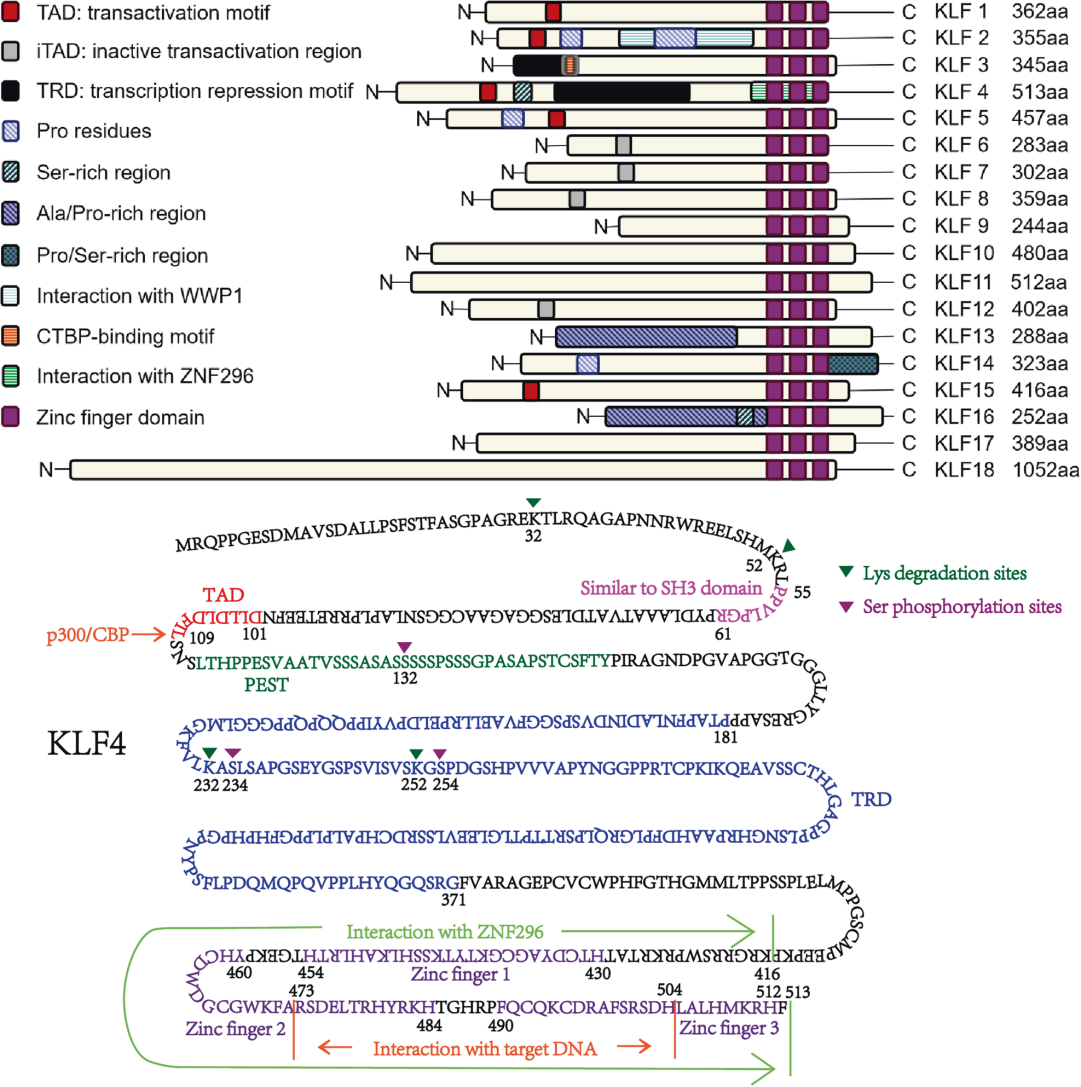
The general proteins structure of human KLF family. All 18 members of the KLFs, among which, KLF1, 2, 4, 5, and 15 contained transactivation motif; KLF3, 6, 7, 8, and 12 contained inactive transactivation domains; KLF3, 4 contained transcriptional repression motif. KLF2, 5, 13, 14, and 16 were rich in Proline; KLF4, 14 and 16 were rich in Serine; KLF13 and KLF16 were rich in Alanine. In addition, KLF2 could bind to WWP1, KLF3 could bind to CTBP, and KLF4 could bind to ZNF296. (aa: amino acid; Data from www.uniprot.org/). Structural sketch of KLF4, which contained the transcriptional activation region (red part), transcriptional repression region (blue part), zinc fingers 1-3 (purple part), and PEST region (green part). In addition, amino acids 473-504 sites were the main sites of KLF4 binding to DNA, amino acids 416-513 could bind ZNF296, and the transcriptional activation region could also bind the transcriptional co-activator P300/CBP. Lys32, 52, 232 and 252 sites of KLF4 protein were the main sites involved in the degradation; Ser132 and Ser254 were the phosphorylation sites that might be associated with KLF4 transcriptional repression, but phosphorylation of KLF4 at Ser234 made KLF4 protein more stable.

The KLF family is composed of at least 18 transcription factors. They are widely distributed in eukaryotes and involved in embryonic development, tissue and organ maturation, stem cell generation, the process of inflammation and tumorigenesis, to name a few [9]. To exert transcriptional regulation, KLFs access to the nucleus via nuclear localization signal (NLS) sequences near or between zinc fingers, and then bind to GC-rich DNA sites via the α-helix and β-folding of zinc fingers structure [10, 11]. The N-terminal transcriptional activation or repression domain binds specifically to the promoters of genes downstream of KLFs, thereby regulating gene transcription [12, 13]. To fulfill these functions, KLFs proteins consist of three parts, the N-terminal domain, the C-terminal domain, and the repetitive amino acids between the N-terminal and C-terminal. First, the N-terminal domain is more variable, enabling KLFs to bind to a wide variety of genes as well as co-activators and co-suppressors, including C-terminal Binding Protein (CtBP) and Sin3A [14]. CtBP is an evolutionarily conserved transcriptional co-suppressor that represses the transcriptional function of genes and negatively regulates the expression of tumor suppressor genes [15]. Differently, Sin3A regulates both transcriptional repression and activation [16]. The C-terminal domain is conserved and the similarity of zinc fingers is more than 65% among the KLFs [17]. The highly conserved nature of the zinc fingers leads to the binding of KLF proteins to the invariant DNA sequence 5’-CACCC-3’ [18]. The three C2H2 residues at the C-terminal are linked by 25, 25 and 23 amino acids, respectively, and each zinc finger recognizes three pairs of DNA bases [19, 20]. Subsequently, a β-β-α structure is formed by α-helix and β-folding [21], which is suitable for binding to the double-helix structure of DNA. The middle part of KLF proteins usually contains a large number of repeated amino acid sequences, forming specific amino acid domains, whereas their unknown functions are yet to be defined. The basic structure of KLFs and its functional domain are shown in Fig. 1.

### General structure of KLF4

Since zinc finger proteins are highly homologous to KLF in the DNA-binding region, KLF4 is classified into the KLFs family [22]. Structurally, the TFIIIA subclass amino acid motif of zinc-finger proteins, Cys-X_2-4_-Cys-X_12_-His-X_3-4_-His, coordinates zinc ions and mediates the binding of the CACCC motifs of target DNA [1, 20, 22]. Interestingly, the zinc-finger of KLF4 usually acts as NLS. KLF4 contains two NLS: one within the zinc fingers, the other in front of the zinc-fingers, an arginine-lysine enrichment region at amino acid positions 384–390 [10]. However, it is not clear whether there is an interaction between the two NLSs affecting the nuclear or cytoplasmic localization of KLF4, especially the role in different types/subtypes of cancers. KLF4 contains a typical proline/serine-rich transcriptional activation domain between amino residues 91 and 117 [23]. Differently, KLF4 also contains a transcriptional repression domain between amino residues 181 and 388 [24], suggesting that KLF4 has a dual regulatory function. Another important domain is PEST (proline-, glutamic-, serine-, threonine-rich domain), which is associated with protein degradation [24]. Mutations in PEST are commonly found in tumors, especially lymphomas and leukemias. It decreases protein ubiquitination and degradation in tumors, leading to sustained activation of the genes [25]. Similarly, PEST of KLF4 also plays a role in protein ubiquitination and degradation [24]. Whether the sustained increase of KLF4 in cancers, especially in early lesions of PDA is associated with the mutations of PEST remains unknown [26]. Finally, PPLPGRP, a 7 amino acid sequence preceding the PEST, is highly similar to the consensus sequence of the SH3 domain and may regulate the signal transduction and cellular function [2]. The amino acid sequence and functional domain of KLF4 are shown in Fig. 1.

### General functions of KLF4

As one of the Yamanaka factors (OSKM, OCT3/4, SOX2, KLF4 and c-MYC), KLF4 is best known for its role in maintaining the stemness of embryonic stem cells and inducing the formation of induced pluripotent stem cell (iPSC) [27, 28]. iPSC formation consists of three components: silencing of the somatic program, activation of stem-cell program, and reorganization of chromatin architecture caused by OSKM. In the mesenchymal-to-epithelial transition of iPSC, KLF4 is responsible for the activation of epithelial marker genes mediating specific interactions at Oct4 loci and recruiting cohesin to the Oct4 enhancer [29–31].

Physiologically, KLF4 critically regulates the epithelial cell differentiation and the development of bone and kidney [32–34]. Embryonic KLF4 deficiency causes altered skin permeability, and the pups show postnatal lethality [35]. KLF4 also decides the fate of cells like other KLFs [36]. Specifically, KLF4 is involved in the proliferation and differentiation of epithelial cells and controls the G1-S transition of the cell cycle after DNA damage through the tumor suppressor gene p53 [24]. Interestingly, the deletion of the second and third zinc fingers of KLF4 reversed the proliferation and differentiation effects on epithelial cells [18]. It may be related to the altered nuclear localization of KLF4 due to incomplete NLS. Moreover, KLF4 regulates hematopoiesis [37], and various neurological disorders, including Alzheimer’s disease, epilepsy, Parkinson’s disease and schizophrenia [38].

Pathologically, KLF4 acts as a context-dependent anti- and pro-inflammatory factor. KLF4 played a protective role in the kidney and antifibrosis role by inhibiting inflammation [39]. The anti-inflammation and antifibrosis may be one of the mechanisms of KLF4 against atherosclerosis [40]. However, KLF4 activated NF-κB signaling pathway and affected the release of pro-inflammatory factors in esophageal keratinocytes [41]. Besides, KLF4 plays an important role in tumorigenesis and development, while KLF4 has a dual effects on tumors [42]. For example, KLF4 played a pro-tumor role in breast cancer maintenance, migration and invasion [43], while in gastrointestinal cancer it acted as a tumor suppressor by inhibiting tumor growth and metastasis [44]. The bidirectional regulation of tumorigenesis is also an aspect that distinguishes KLF4 from most KLFs, probably due to the different activities of the KLF4 transcriptional repression or activation domains in different tumor types/subtypes.

## The unique roles of KLF4 in tumors

KLF4 plays an important role in the development and progression of various types of cancers, including lung, breast, colorectal and pancreatic cancers. However, whether KLF4 is cancer-promoting or cancer-suppressing remains a mystery. In general, the dual effect of KLF4 on cancers depend on the type/subtype of cancer and the molecular regulation in signaling pathways. KLF4 acted as a tumor suppressor in gastrointestinal tumors [45] and cutaneous squamous cell carcinoma, and as a pro-cancer factor in cutaneous melanoma and breast cancer [7, 46, 47]. In addition, the effects of KLF4 vary from the stages of cancers. In the case of PDA, KLF4 protein was increased in early lesions of PDA, including ADM and PanIN, and promoted the formation of pre-cancerous pancreatic lesions in the mouse model [26, 48]. In contrast, KLF4 was decreased in human PDA and human PDA cell lines [49]. KLF4 also plays a positive role in inhibiting tumor proliferation, epithelial-to-mesenchymal transition (EMT), and metastasis [50–52]. Therefore, the anti- or pro-cancer roles of KLF4 are unpredictable [53]. However, the mechanisms underlying the altered expression and function of KLF4 remain unclear. The following discussion focuses on the specific cancer-regulating roles of KLF4 in ten common cancers. The effects of KLF4 on various tumor types are summarized in Table 1.

**Table 1:** The roles of KLF4 in ten common tumors according to the mortality rate of Clinicians Cancer Statistics in 2021.

Tumor types	Tumor subtypes	KLF4 function	Molecules regulated by KLF4	Molecules regulate KLF4	References
Lung cancer	Non-small cell lung cancer	promote & suppress tumors	PLAC8 [S1] JNK [S2] MMP2 [S3] p21, cyclinD1 [S4]	HDAC3 [S5] UCHL3 [S6] Numb [S7] USP10 [S8]	[S1-S10]
	Small cell lung cancer	suppress tumors			
Breast cancer	Primary ductal carcinoma	promote tumors	Snail [S11] estrogen receptor-α [S12] epidermal growth factor receptor [S13] miR-206 [S14] cycle-related protein [S15] p53 [S16] CXCL5 [S17]	KDM7a [S18] PRMT5 [S19] AtXN3 [S20] miR-10b [S21] SIRT1 [S22] miR-7 [S23] FBXO32 [S24]	[S11-S26]
	Triple-negative breast cancer	suppress tumors			
Prostate cancer	Developing prostate cancer	promote tumors	p21, p27, p57, CCNB1 [S27]	SLUG [S28] DNMT1 [S29] KMT2D [S30] LINC00673 [S31] androgen receptor [S32] miR-7 [S33] miR-148-3p, miR-152-3p [S34]	[S27-S38]
	Advanced prostate cancer	suppress tumors			
Colorectal cancer	–	suppress tumors	GINS4 [S39] TGF-β1 [S40] IFITM3 [S41] cyclinD1, p21, NDRG2 [S42] p53 [S43]	miR-103/107 [S44] miR-29a [S45] miR-25-3p [S46] miR-92a [S47] miR-10b [S48]	[S39-S50]
Pancreatic cancer	Precancerous lesions	promote precancerous lesions	MSI2 [S51] CD44 [S52] Caveolin-1 [S53] p27Kip1 [S54]	DNMT1 [S55] MUC5AC [S56] PPARγ [S57]	[S51-S61]
	Ductal adenocarcinoma	suppress tumors			
Hepatocellular carcinoma	–	suppress tumors	TGF-β [S62] vitamin D receptor [S63] TIMP-1 and TIMP-2 [S64] miR-31 [S65] monoglyceride lipase [S66] CD9 and CD81 [S67] P-cadherin [S68] SIRT4 [S69]	SET8 [S69] DDX17 [S70] miR-9-5p [S71] TRAF7 [S72]	[S62-S73]
Ovarian cancer	Advanced epithelial ovarian cancer	suppress tumors	TGF-β, E-cadherin [S74] Bcl2/Bax [S75]	LINC01210 [S76] CircPLEKHM3 [S77] SIRT1 [S78]	[S74-S78]
	Other ovarian cancer				
Esophageal cancer	Esophageal squamous cell carcinoma	promote & suppress tumors	survivin [S79] Keratin 13 [S80]	miR-10b [S81] miR-92B-3p [S82] CircRNA 7 [S83] miR-7-5p [S84]	[S79-S86]
	Advanced cancer	promote tumors			
Bladder cancer	Uroepithelial carcinoma	suppress tumors	twist1 [S87]	promoter methylation [S88] miR-10b [S89] METTL3/YTHDF2 [S90] NOTCH-1 [S91] HDAC2, CBP/p300 [S92]	[S87-S93]
	EMT	promote invasion/EMT			
Brain cancer	Meningioma Medulloblastoma	suppress tumors	HIF [S94] MMP9, Bcl2 [S95] cyclinD2 [S96] cyclinD1 [S97] guanine nucleotide exchange factors [S98]	–	[S94-S99]
	Glioblastoma	promote tumor cells activity			

### Lung cancer

Lung cancer is a lethal human disease with the second highest number of new cases [54]. KLF4 negatively regulates the lung cancer. In fact, KLF4 inhibited the growth, migration, invasion, metastasis and EMT of lung cancer cells through SIRT6/Snails/KLF4, KLF4/Plac8, Numblike-KLF4, KLF4-MMP2 and c-Jun-NH2-terminal kinase signaling pathway [55–59]. Importantly, KLF4 inhibited tumor growth by regulating the typical cyclin regulatory molecules, p21 and cyclinD1 [60]. Clinically, KLF4 is significantly lower in lung cancer than in normal lung tissue [61]. However, some studies suggested that KLF4 promoted the progression of non-small cell lung cancer (NSCLC) and this function of KLF4 was related to its subcellular localization [62]. Localization of KLF4 in the nucleus was associated with a poor prognosis, while localization of KLF4 in the cytoplasm had a better prognosis. The different subcellular localization of KLF4 may link to the different isomers of KLF4, *i.e*., wild-type KLF4 is expressed in the nucleus, whereas the isomer KLF4α is localized in the cytoplasm, and they indeed exhibit the opposite effects [63].

### Breast cancer

Breast cancer is the second lethal cancer in women, with the highest number of new cases [54]. The effect of KLF4 on breast cancer is “controversial”. In human primary ductal carcinoma of the breast, KLF4 promotes tumor progression and the localization of KLF4 in the nucleus relates to poor prognosis [64]. However, in the highly malignant triple-negative breast cancer, high expression of KLF4 inhibits cancer cells proliferation and invasion, and KLF4 is also a marker of prognosis in triple-negative breast cancer [65, 66]. The discrepancy maybe related to alterations in major signaling pathways caused by different cancer types/subtypes. KLF4 inhibited the breast cancer cells growth, metastasis, and invasion through inhibition of Snail, estrogen receptor, and epidermal growth factor receptor [67–69]. In contrast, knockdown of SIRT1 downregulated KLF4 and inhibited the stemness of breast cancer cells [70]. KLF4 inhibited breast cancer cell apoptosis through the p53-KLF4-p21-cyclinD1 axis [6] and KLF4 deficiency inhibited breast cancer growth and lung metastasis [71]. Therefore, KLF4 may promote the progression of low malignancy primary ductal carcinoma of the breast, whereas KLF4 inhibits the progression of high malignancy triple-negative breast cancer. The underlying mechanisms of non-invasive carcinomas as well as other types of invasive carcinomas warrant further investigations.

### Prostate cancer

KLF4 generally inhibits the development of prostate cancer. For instance, the expression of KLF4 is decreased in primary and metastatic prostate cancer tissues. Preclinical studies have also verified that KLF4 inhibits the growth and migration of prostate cancer cells [72]. Further studies have found that androgen receptors effectively activate KLF4 and reduce the proliferation, invasion and bone metastasis of prostate cancer cells [73]. Conversely, downregulation of KLF4 enhanced the aggressiveness of prostate cancer [74]. In addition, certain signaling pathways such as TGF-β promoted the growth, metastasis and invasion of prostate cancer by directly downregulating KLF4 expression [75, 76]. However, a few reports also suggested that KLF4 was associated with the stability of tumor stem cells [77]. Some non-coding RNAs such as miR-148-3p and miR-152-3p inhibited prostate cancer progression by downregulating KLF4 expression [78]. This seemingly opposite effect in prostate cancer may be related to the subcellular localization of KLF4, *i.e*., KLF4 was usually expressed in the cytoplasm of highly malignant prostate cancers [79].

### Colorectal cancer

The antitumor role of KLF4 in colorectal cancer is evidently clear. KLF4 was downregulated in human colorectal cancer tissues as compared with normal mucosa [80]. KLF4 acted as a tumor suppressor to inhibit the colorectal cancer growth in mice [81]. KLF4 inhibited colorectal cancer cell proliferation through upregulating p21 and NDRG2 (a molecule downstream of Myc), and downregulating cyclinD1 (the key molecule of cell cycle G1/S extension), GINS complex subunit 4 (GINS4), and IFITM3 (a tumor metastasis-associated molecule) [80, 82, 83]. Conversely, KLF4 deficiency accelerated the progression of colitis and colon cancer [84]. There were multiple microRNAs downregulating KLF4, including microRNA-92a and microRNA-10b, promoting colorectal cancer growth, migration, proliferation, and metastasis [85, 86]. KLF4 deficiency is also associated with tumor development by EMT [87]. These evidences elucidate an antitumor role of KLF4 as a tumor suppressor in colorectal cancer.

### Pancreatic cancer

Although the incidence of PDA is not as high as that of breast, lung and colorectal cancers, the mortality rate is almost as high as the morbidity rate due to the lack of effective treatment. The current measures for PDA management have shifted to early diagnosis and treatment. In PDA, KLF4 promoted the formation of precancerous lesions in mouse models, including ADM and PanIN. Human PDA was associated with chronic pancreatitis, in which ADM was observed [88], and ADM regulated the formation and progression of PanIN and PDA [89, 90]. Notably, KLF4 was increased in ADM and PanIN in mouse models and played a key role in the formation of ADM and PanIN. Knockdown of KLF4 significantly slowed down the formation of ADM and PanIN, while overexpression of KLF4 promoted the formation and progression of AMD and PanIN [91, 92].

However, KLF4 inhibits cancer cell proliferation, EMT, invasion and metastasis in the advanced stage. PDA had a significantly reduced KLF4 expression as compared to normal pancreatic tissues and benign pancreatic lesions [93]. KLF4 inhibited PDA proliferation and metastasis through multiple pathways, including inhibition of the potential tumor protein Musashi 2 (MSI2), the stem cell-associated protein CD44, and the promotion of the cell cycle-dependent protein kinase inhibitor p27Kip1 [50, 52, 94]. In addition, KLF4 inhibited the growth of PDA by suppressing aerobic glycolysis and activation of KLF4 inhibited the PDA cells proliferation via peroxidase ligands [50, 95]. KLF4 also inhibited PDA cell EMT by downregulating Caveolin-1, which was closely associated with tumor metastasis [51]. Evidently, KLF4 promotes the early lesions of PDA but inhibits the development of advanced PDA. Differences in the cancer types/subtypes may render KLF4 diverse roles in different stages of PDA [63]. Therefore, there is sufficient evidence for an inhibitory role of KLF4 in PDA, whereas its role in early PDA lesion needs validation clinically.

### Hepatocellular carcinoma

KLF4 plays a role in inhibiting the development of hepatocellular carcinoma (HCC). KLF4 expression is lower in HCC tissues than in adjacent tissues, and high KLF4 expression is considered as a marker of benign prognosis after surgical resection [96]. In contrast, deletion or downregulation of KLF4 promotes HCC progression [97]. Mechanistically, KLF4 inhibits the progression of HCC by regulating a large number of signaling pathways. For example, KLF4 inhibited HCC progression by promoting KLF4-p-cadherin-GSK-3β and KLF4-CD9/CD81-JNK [98, 99] signaling pathways. KLF4 upregulated monoglyceride lipase in the metabolic pathway and inhibit HCC cell migration [100]. KLF4 also inhibited the migration and invasion of tumor cells by suppressing biological enzymes, including TIMP-1 and TIMP-2 [101]. Monomethyltransferase SET8 can directly inactivate KLF4 and promote tumor progression [102]. In human primary and lymphatic metastatic HCC samples, KLF4 negatively correlated with vitamin D receptor and KLF4 inhibited HCC progression by downregulating vitamin D receptor [103]. Therefore, KLF4 consistently inhibits the growth and progression of HCC.

### Ovarian cancer

Ovarian cancer can be divided into epithelial ovarian cancer (EOC) and non-epithelial ovarian cancer, with the former accounting for more than 95% of ovarian malignancies [104]. KLF4 has an inhibitory effect on ovarian cancer, but inconclusive. KLF4 transcript levels are downregulated in human advanced EOC samples as compared to normal ovarian tissue. Inactivation of KLF4 is frequently found in ovarian cancer patients [105]. However, the reason for KLF4 inactivation in human ovarian cancer samples is unclear. Overexpression of KLF4 reduced ovarian cancer cell proliferation, migration and invasion by inhibiting TGF-β-induced EMT [106]. The signaling axis of BRCA1/DNAJB6/KLF4/AKT1 regulated ovarian cancer progression [107]. Besides, SIRT1 and LINC01210 downregulated KLF4, thereby promoting the proliferation, migration and invasion of ovarian cancer cells [108, 109]. However, more evidence from mouse and human ovarian cancer cells is needed to validate the correlation between KLF4 and EOC. There is a lack of definitive evidence whether and how KLF4 affects ovarian cancer development and progression, especially in patients. Therefore, the role of KLF4 as an antitumor factor in ovarian cancer needs more in-depth study.

### Esophageal cancer

More than 90% of esophageal cancers are squamous cell cancers (SCC) [110]. The antitumor effect of KLF4 on esophageal squamous cell cancers (ESCC) is uncertain. KLF4 is reduced in human ESCC as compared to normal tissue. A few studies have suggested that KLF4 is reduced in early-stage of tumors but increased in invasion stage, and the level of KLF4 do not significantly correlate with the survival in ESCC [111]. Other study found that KLF4 inhibited the proliferation and invasion of tumor cells and suppressed the progression of ESCC [112]. The inhibitory effect of KLF4 on cancer cells proliferation may be related to the suppression of survivin [113]. Also, KLF4 could promote keratin 13 for squamous cell carcinoma differentiation [114]. Conversely, upregulation of KLF4 was associated with inflammation and esophageal carcinoma formation, while miR-7 could inhibit the migration and invasion of ESCC through downregulation of KLF4. KLF4 could induce the initiation of ESCC by promoting inflammation without increasing its expression [115, 116]. Nonetheless, it is clear that there is a negative correlation between KLF4 and ESCC progression, while the antitumor role of KLF4 and its molecular mechanisms in early and advanced stages of ESCC need further investigation.

### Bladder cancer

Bladder cancer (BC) can be divided into uroepithelial carcinoma of the bladder, squamous carcinoma of the bladder, adenocarcinoma of the bladder and bladder sarcoma (UBC). UBC is the most common type with an incidence of over 90%. Limited data indicated that KLF4 inhibited the development of BC, while KLF4 was downregulated in a variety of UBC tissues and cells as compared to normal bladder epithelial tissues and cells. Overexpression of KLF4 inhibited BC cells growth and induced apoptosis [117]. Mechanistically, epigenetic modifications such as promoter methylation and deacetylation inactivated KLF4 and promoted BC proliferation, progression and recurrence [118, 119]. In addition, miR-10b bound directly to KLF4 and promoted BC migration and invasion [120]. Moreover, KLF4 mRNA can be downregulated by the METTL3/YTHDF2 m6A axis and Notch-1, thereby promoting the BC formation [121, 122]. In contrast, data from 398 UBC patients showed that KLF4 was correlated with EMT and poor prognosis in advanced UBC, and Twist1 was an important target of KLF4 [123]. In other subtypes of BC, KLF4 may inhibit tumor progression in squamous cell carcinoma or adenocarcinoma. Therefore, KLF4 appears to be negatively associated with bladder cancer progression, and paradoxically, KLF4 may play a facilitating role in advanced BC.

### Other tumors

The incidence of brain cancer accounts for approximately 1–3% of all human tumors. The most common benign brain cancers are pituitary tumors and meningiomas, while the most common malignant brain cancer is glioma. Clinical meningioma is frequently associated with KLF4 mutations and low expression of KLF4 RNA and protein [124, 125]. Inactivation of KLF4 is related to the CpG island methylation of the KLF4 promoter [125]. Further studies have revealed that the KLF4 K409Q mutation exhibits enhanced hypoxic signaling and is involved in the adaptation of tumor cells to the anaerobic environment [126]. As a tumor suppressor, KLF4 affects apoptosis, proliferation, invasion and cell cycle of tumor cells during the malignant development of meningioma [127]. In glioma, the rapid proliferation of tumor cells often leads to local nutritional deficiency. However, KLF4 can alter the cells cycle processes through G2/M phase arrest, protect cells from nutrient deprivation-induced death, and promote the development of glioblastoma. This process is associated with the guanine nucleotide exchange factor, induction of mitochondrial fusion, cell cycle arrest and cytoprotective effectors [128]. Currently, the role of KLF4 in different subtypes of brain cancer is yet to be defined. KLF4 may also play a regulatory role in cutaneous squamous cell carcinoma, lymphoma, leukemia and cervical cancer [46, 129–133]. However, the evidence for anti- or pro-tumor effects of KLF4 is not conclusive.

In summary, as a transcription factor, KLF4 has a broad regulatory role in tumors and its signaling pathways are mainly involved in cell cycle and proliferation, *e.g*., p53, p21 and cyclin-D. The inconsistent roles of KLF4 in tumor development and progression may be due to multiple factors, including the types of tumors, stage of tumor progression, isomers and their regulation. The different roles of KLF4 are best exemplified in PDA, where KLF4 plays opposing role at different stages of PDA development and progression. Tumor subtypes clearly dictate the regulatory roles for KLF4 as exemplified by the evidence that KLF4 is more likely to promote tumor development from endothelial and squamous cells, while the opposite role in others. Therefore, the function of KLF4 in tumorigenesis is highly complex, given the context of different tumor subtypes, diverse molecular regulation and malignancy of tumor cells. Therefore, the signaling pathways of KLF4 are summarized next in an attempt to understand the dual regulatory role of KLF4 on tumors.

## Signaling pathways of KLF4 in tumors

KLF4 regulates a large number of genes and biological factors involved in proliferation, differentiation and apoptosis, and crucially regulates the development and progression of various types of tumors [134]. In PDA, KLF4 promotes the development of PDA by facilitating the formation of ADM and PanIN or directly through IPMNs [26, 135]. Mechanistically, KLF4 regulates PDA progression in numerous signaling pathways by directly binding to the downstream effectors or their gene promoters. Importantly, KLF4 played a role in cell differentiation and cytoskeletal organization by binding directly to p53, keratin 19, the β-catenin/LRH-1 complex, and the oncofetal RNA-binding protein LIN28B [136–138]. There were several isomers of KLF4, among which KLF4α was present in the cytoplasm and promoted PDA progression [139]. SATB2 promoted PDA progression by upregulating the stem cell markers, CD44, CD24, and CD133, which was related to the regulation of KLF4 [140]. In PanIN, secreted mucin 5AC (MUC5AC) stimulated PDA stem cell regeneration by upregulating KLF4 through integrin αVβ5, pSrc (Y416) and pSTAT3 (Y705) [92]. Thus, MUC5AC promoted PDA development and progression, while KLF4 only acted as an indirect downstream molecule of MUC5AC.

Generally, KLF4 inhibits PDA progression by binding to various promoters of genes related to proliferation, *e.g*., p21, p27^Kip1^, CD44, MSI2, Caveolin-1, and FOXM1 [50–52, 94, 95, 141]. KLF4 also inhibits tumor development by regulating metabolism-related molecules, including LDHA and suppressing aerobic glycolysis pathways [142]. Pharmocologically, KLF4 can be downregulated by gemcitabine, while the knockdown of KLF4 enhances ZEB1 expression and gemcitabine resistance [143]. In addition, dietary 3,3’-diindolylmethane inhibited PDA development by downregulating DNMT1 and suppressing KLF4 promoter methylation [49]. Conversely, α-mangiferin inhibited KLF4, upregulated E-cadherin, and suppressed EMT via Hedgehog and Nanog signaling pathways. Interestingly, α-mangiferin inhibited the expression of N-cadherin, Snail and Slug, and downregulated the mesenchymal phenotype and suppressed the ability of PDA stem cells to invade and metastasize, which was also related to the regulation of KLF4 expression [144].

**Fig. 2:**
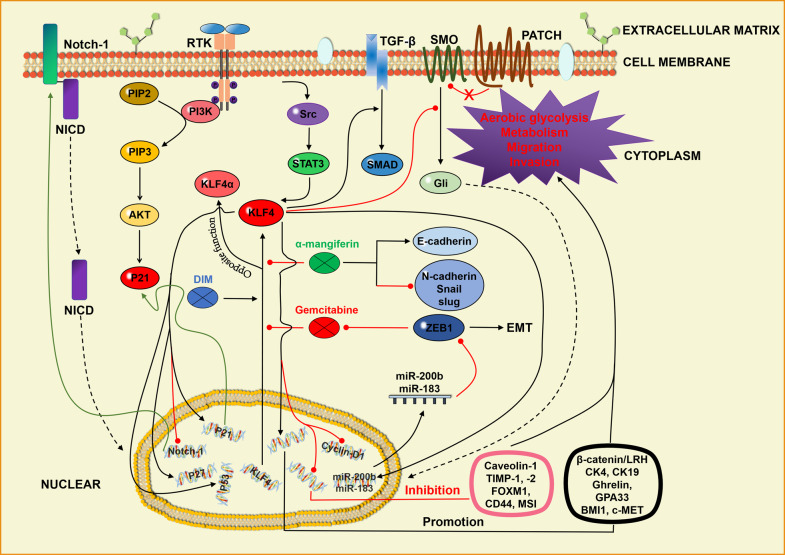
Molecular mechanism of KLF4 regulating the tumors progression. KLF4 exerted its tumor regulatory role mainly by promoting PI3K-AKT-P21, TGF-β and repressing NOTCH-1 and SHH signaling pathways. KLF4 promoted the expression of P21, P27, P53, β-catenin/LRH, CK4, CK19, Ghrelin, GPA33, BMI1 and c-MET mainly through transcriptional activation, while repressing the expression of Cyclin-D1, Caveolin-1, TIMP-1, TIMP-2, FOXM1, CD44 and MSI, exerting a tumor-regulatory effect. Among them, P21, P27, P53 and Cyclin-D1 were currently considered to be the most important downstream genes of KLF4. In addition, the isomer of KLF4, KLF4α, played an opposite role to that of KLF4. Black arrows represented promoting effects, while red circular arrows represented repressing effects.

Finally, KLF4 also regulates the development and progression of several other cancers by directly regulating cell proliferation and differentiation factors, including CyclinD1, p53, Keratin4, and Keratin19 [24]. KLF4 also regulates TGF-β, Notch-1 and P57, affecting the tumor proliferation, differentiation and apoptosis [5]. Other KLF4-regulated factors include Ghrelin, TIMP-1 and TIMP-2, ABL, BMI1, Met, GPA33 and Hsp90, which are associated with tumor development and metastasis [5]. Among all of them, p53, p21 and cyclin-D are the main targets of KLF4 [134]. The signaling pathways of KLF4 expression and regulation in PDA and other tumors are summarized in Fig. 2.

## KLF4 as a biomarker and therapeutic target for cancers

### KLF4 as a biomarker for cancers

Numerous studies have demonstrated that KLF4 inhibits the progression of colorectal, gastric, and hepatocellular carcinomas, and low KLF4 expression is clearly related to poor overall survival. Importantly, the higher the malignancy of tumor, the lower the expression of KLF4 [134]. This phenomenon is also supported by analyses of clinical data, a total of 2988 patients to assess the prognostic value of KLF4 in solid tumors [145]. Thus, KLF4 is expected to be a diagnostic and prognostic biomarker for colorectal, gastric and hepatocellular carcinomas.

Although KLF4 inhibits the tumor proliferation and metastasis in most cases of SCLC and NSCL, KLF4 expression is higher in some NSCL than in normal tissue, which implies a positive association between KLF4 and NSCL [146]. In most highly malignant triple-negative breast cancers, KLF4 prevents tumor proliferation, migration, and invasion, and is a biomarker of benign prognosis. However, loss of KLF4 actually inhibits the growth and metastasis of ductal adenocarcinoma of the breast. In prostate cancer, KLF4 appears to inhibit the progression of advanced prostate cancer, but promotes the development of early prostate cancer. Also, KLF4 suppresses tumor progression in ovarian cancer, melanoma, leukemia [147] and cervical cancer. In contrast, KLF4 promotes the tumor progression in squamous cell carcinoma of the head and neck, osteosarcoma, and glioblastoma. Therefore, the meaningful role of KLF4 as a diagnostic and prognostic biomarker needs further confirmation in these tumors.

PDA cells can originate from a variety of cells in the pancreas, such as acinar cells, ductal cells, central acinar cells, endocrine cells and their progenitor cells, but not from a single cell type [148–153]. Tissue microenvironment, such as injury and inflammation, impacts the formation of PDA precursor cells by mutations in proto-oncogenes and/or tumor suppressor genes, *e.g*., *KRAS* and *TP53* [154]. Susceptibility to tumor formation varies by cell types and is also relates to the heterogeneous functional status of mutated genes [155, 156]. In human and mouse PDA, protein levels of KLF4 are lower than normal pancreas. KLF4 expression decreases with increasing tumor malignancy [93]. Downregulation of KLF4 affects multiple important signaling pathways, including NOTCH [157]. During the tumor metastasis, KLF4 is further downregulated [94], whereas downregulation of KLF4 is associated with poor prognosis in PDA. Therefore, KLF4 can be an independent prognostic marker for PDA.

ADM is an important precancerous lesion of PDA, in which KLF4 is upregulated with *KRAS* mutations, caerulein treatment and pancreatic duct ligation [26]. KLF4 inhibits the NOTCH signaling pathways and promotes transdifferentiation of acinar cells to duct-like cells by acting on its downstream targets such as p53, p27, p21and Cyclin-D [50, 158–161]. Given the key role of KLF4 in the formation of ADM, KLF4 can be considered as one of the biomarkers of ADM [26]. ADM can further develop into PanIN and progresses to PanIN-3 as carcinoma in situ, and consistently, KLF4 gradually increases in PanIN [26, 162]. Thus, KLF4 may be used as a precancerous biomarker for early diagnosis of PDA. However, it is a huge challenge to detect biomarkers for early PDA lesions clinically, especially in organs like the pancreas. Nonetheless, KLF4 helps to evaluate PDA development and progression, determine its malignancy after diagnosis and predict prognosis after treatment.

**Fig. 3:**
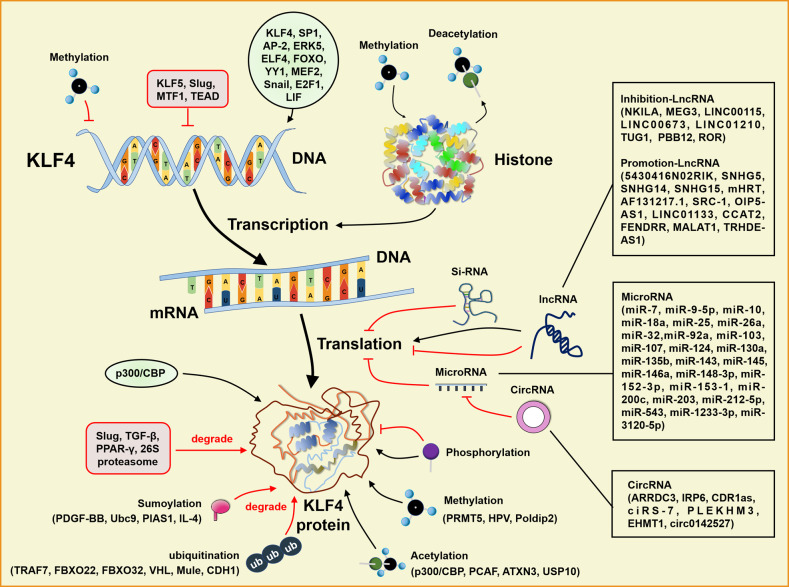
The molecular regulation of KLF4 expression. The figure mainly showed the mechanism diagram of KLF4 transcriptional and translational processes that regulated its expression, including transcriptional regulation, post-transcriptional regulation, and post-translational regulation. At the level of the KLF4 gene, the transcription factors KLF4, SP1, AP-2, ERK5, LIF, ELF4, FOXO, MEF2, Snail, E2F1 and YY1 targeted to bind KLF4 and promoted the KLF4 transcription, while KLF5, Slug, MTF1 and TEAD inhibited the transcription. Besides, KLF4 DNA methylation inhibited the transcription, while the histone methylation and deacetylation entangled by KLF4, promoted KLF4 transcription at the epigenetic level. In the process of KLF4 translation, KLF4 was chiefly regulated by microRNAs and lncRNAs, in which siRNA and microRNAs targeted to bind KLF4 mRNA and inhibited the KLF4 translation, while circRNAs mitigated the KLF4 inhibition by binding microRNAs. However, a small proportion of IncRNAs could promote the KLF4 translation, while most LncRNAs inhibited the KLF4 translation. After the KLF4 protein was successfully translated, it was also regulated by a number of molecules. Protein-protein interactions included co-activation of P300 /CBP and degradation of Slug, TGF-β, PPAR-γ and 26 S Proteasome. Moreover, protein modifications played a dominant role in the regulation of KLF4 activity: the methylation and de-sumoylation of KLF4 stabilized the protein structure; acetylation and de-ubiquitination play activation roles; ubiquitination and sumoylation contribute to degradation.

In other tumors, KLF4 remains “complex” as a molecular marker for diagnosis and prognosis. There are many KLF4 downstream molecules, certain of which can play a dominant role in certain types or subtypes of tumors [163]. Therefore, a combination of analysis of crosstalk between KLF4 and its downstream can help to accurately define the role of KLF4 in different tumors and to determine whether KLF4 can be used as a criterion for early diagnosis and prognostic assessment. The molecular mechanism of KLF4 regulation of tumorigenesis development is shown in Fig. 3.

### KLF4 as a therapeutic target for cancers

KLF4 acts as a context-dependent antitumor or tumor-promoting factor, holding limited potential as a therapeutic target. However, few compounds have been found to act directly on KLF4. Sulforaphane, a natural compound derived from broccoli, inhibited the progression of colon cancer by inducing KLF4 and enhancing the KLF4-p21 signaling [164]. Kenpaullone, a potent inhibitor of CDK1/cyclin B and GSK-3β, inhibited the proliferation and migration of breast cancer cells and induced cell death by downregulating KLF4 [165]. The two compounds exert antitumor effects in vitro, which needs validation in vivo, especially Kenpaullone [166].

Certain drugs also regulate KLF4 expression through epigenetic modifications. Decitabine, the most potent inhibitor of gene methylation, upregulated KLF4 in renal fibrosis by inhibiting the methylation of KLF4 promoter, which contributed to the inhibition of EMT [167]. Small molecule inhibitors WX2-43 and MM-102 also inhibited KLF4 methylation and destabilized KLF4 by acting on methyltransferases to inhibit the progression of triple-negative breast cancer [168, 169]. Interestingly, higher levels of aliphatic acid in the obese state led to an increase in DNMT1 and DNMT3, and raised the methylation level of KLF4 promoter [170]. All-trans retinoic acid increased KLF4 activity by phosphorylated KLF4 via MAPK, promoted P300 binding to KLF4 [171]. Pioglitazone, a PPAR-γ agonist, stabilized KLF4 protein by activating AKT signaling and reducing KLF4 ubiquitination [172]. However, clinical researches of WX2-43, MM-102 and Pioglitazone have not been reported.

Currently, a few compounds have been designed to target the tumor-regulating effects of KLF4, and are available for clinical trials. APTO-253, a targeted inducer of KLF4, have been found in a clinical trial to have successfully halted the progression of some advanced tumors, especially leukemia [173]. However, the company indicated that this drug inhibited MYC and had discontinued the clinical development. In a clinical trial, metformin had a positive effect on colorectal cancer and endometrial cancer by increasing KLF4 expression [146]. Also, KLF4 can be negatively regulated by the cis-structural transcription factor CDX2. In colon cells, CDX2 upregulated KLF4 by binding to KLF4 promoter and reducing the association of histone 3 lysine 4 trimethylation with KDM5B demethylase, and acting as a tumor suppressor. However, transcription factors are generally considered undruggable. Therefore, it is premature to consider KLF4 as a general target for cancer therapy.

## Conclusions and future directions

KLF4 expression is finely orchestrated during embryonic development and physiology [1]. Understanding the role of KLF4 in different organs can help to elucidate its function in pathological processes, including inflammation and tumorigenesis. It is known that KLF4 can regulate tumorigenesis and this role can be further complicated by tumor types and stages. Given its complex role in tumor biology, it is difficult to universally define KLF4 as a therapeutic target for all types of tumors. However, it is also tempting to use KLF4 as a biomarker for tumor diagnosis and prognosis, which must be in conjunction with the knowledge of physiology, pathology, and imaging data for specific types/subtypes of tumors. In some tumors, analysis in combination with important downstream molecules of KLF4 will yield more information assisting cancer diagnosis and prognosis. However, KLF4 has not been detected in body fluid samples. Therefore, a diagnostic approach to detect KLF4 from body fluids seems somewhat impractical. Whether KLF4 can signal between tumor cells via exosomes remains a mystery [174], although exosomes are found to contain pluripotency genes (OCT4, SOX2, KLF4, C-Myc and Nanog) during embryonic development [175]. Further studies are needed to determine whether KLF4 is enriched in tumor exosomes and whether exosomal KLF4 can be used for clinical diagnosis and therapy.

KLF4 regulates a large number of genes related to cell proliferation and differentiation. Expression of KLF4 has a significant impact on the formation of precancerous lesions, such as ADM and PanIN [24]. In conjunction with the current new strategies in oncology treatment, 90% of tumors can be treated radically when detected at an early stage. Therefore, small molecule inhibitors or combination chemotherapeutic agents designed to target KLF4 protein in precancerous lesions may have an effective role in cancer prevention and intervention. In the future, a comprehensive understanding of the KLF4 signaling and its molecular mechanisms during precancerous lesion formation in various tissues will not only help to identify and define whether KLF4 is a “tumor suppressor” or a “tumor promoter,” but also serve as a basis for designing appropriate and effective intervention strategies against PDA and even the other tumors.

## Supplementary information

Supplementary references for Table 1

## Data Availability

The corresponding author will provide the relevant data used to support the opinions and conclusion of this review upon reasonable request.
