# Supplementary material for: KLF4 transcription factor in tumorigenesis
Source: Cell Death Discov. 2023 Apr 8;9:118. doi: 10.1038/s41420-023-01416-y (PMC10082813; doi:10.1038/s41420-023-01416-y)
Supplement: Supplementary file 1 — Supplementary references for Table 1 [file 41420_2023_1416_MOESM1_ESM.pdf]

Supplemental References for Table 1

[S1] Jia Y, Ying X, Zhou J, Chen Y, Luo X, Xie S, Wang QC, Hu W, Wang L. The novel KLF4/PLAC8 signaling pathway regulates lung cancer growth. Cell Death Dis. 2018 May 22;9(6):603. doi: 10.1038/s41419-018-0580-3. PMID: 29789534; PMCID: PMC5964121.

[S2] Wu Y, Lin L, Wang X, Li Y, Liu Z, Ye W, Huang W, Lin G, Liu H, Zhang J, Li T, Zhao B, Lv L, Li J, Wang N, Liu X. Overexpression of Krüppel-Like Factor 4 Suppresses Migration and Invasion of Non-Small Cell Lung Cancer Through c-Jun-NH2-Terminal Kinase/Epithelial-Mesenchymal Transition Signaling Pathway. Front Pharmacol. 2020 Jan 8;10:1512. doi: 10.3389/fphar.2019.01512. PMID: 31969824; PMCID: PMC6960180.

[S3] Li S, Huang L, Gu J, Wu J, Ou W, Feng J, Liu B, Xu X, Zhou Y. Restoration of KLF4 Inhibits Invasion and Metastases of Lung Adenocarcinoma through Suppressing MMP2. J Cancer. 2017 Sep 27;8(17):3480-3489. doi: 10.7150/jca.21241. PMID: 29151932; PMCID: PMC5687162.

[S4] Hu W, Hofstetter WL, Li H, Zhou Y, He Y, Pataer A, Wang L, Xie K, Swisher SG, Fang B. Putative tumor-suppressive function of Kruppel-like factor 4 in primary lung carcinoma. Clin Cancer Res. 2009 Sep 15;15(18):5688-95. doi: 10.1158/1078-0432.CCR-09-0310. Epub 2009 Sep 8. PMID: 19737957; PMCID: PMC2745510.

[S5] Yu T, Chen X, Zhang W, Liu J, Avdiushko R, Napier DL, Liu AX, Neltner JM, Wang C, Cohen D, Liu C. KLF4 regulates adult lung tumor-initiating cells and represses K-Ras-mediated lung cancer. Cell Death Differ. 2016 Feb;23(2):207-15. doi: 10.1038/cdd.2015.85. Epub 2015 Jun 26. PMID: 26113043; PMCID: PMC4716302.

[S6] Ouyang L, Yan B, Liu Y, Mao C, Wang M, Liu N, Wang Z, Liu S, Shi Y, Chen L, Wang X, Cheng Y, Cao Y, Xiao D, Zhang L, Liu S, Tao Y. The deubiquitylase UCHL3 maintains cancer stem-like properties by stabilizing the aryl hydrocarbon receptor. Signal Transduct Target Ther. 2020 Jun 17;5(1):78. doi: 10.1038/s41392-020-0181-3. PMID: 32546741; PMCID: PMC7297794.

[S7] Vaira V, Faversani A, Martin NM, Garlick DS, Ferrero S, Nosotti M, Kissil JL, Bosari S, Altieri DC. Regulation of lung cancer metastasis by Klf4-Numb-like signaling. Cancer Res. 2013 Apr 15;73(8):2695-705. doi: 10.1158/0008-5472.CAN-12-4232. Epub 2013 Feb 25. PMID: 23440423; PMCID: PMC3695627.

[S8] Wang X, Xia S, Li H, Wang X, Li C, Chao Y, Zhang L, Han C. The deubiquitinase USP10 regulates KLF4 stability and suppresses lung tumorigenesis. Cell Death Differ. 2020 Jun;27(6):1747-1764. doi: 10.1038/s41418-019-0458-7. Epub 2019 Nov 20. PMID: 31748695; PMCID: PMC7244734.

[S9] Liu M, Li X, Peng KZ, Gao T, Cui Y, Ma N, Zhou Y, Hou G. Subcellular localization of Klf4 in non-small cell lung cancer and its clinical significance. Biomed Pharmacother. 2018 Mar;99:480-485. doi: 10.1016/j.biopha.2018.01.090. Epub 2018 Feb 20. PMID: 29665649.

[S10] Li Z, Huang J, Shen S, Ding Z, Luo Q, Chen Z, Lu S. SIRT6 drives epithelial-to-mesenchymal transition and metastasis in non-small cell lung cancer via snail-dependent transrepression of KLF4. J Exp Clin Cancer Res. 2018 Dec 22;37(1):323. doi: 10.1186/s13046-018-0984-z. PMID: 30577808; PMCID: PMC6303940.

[S11] Yori JL, Seachrist DD, Johnson E, Lozada KL, Abdul-Karim FW, Chodosh LA, Schiemann WP, Keri RA. Krüppel-like factor 4 inhibits tumorigenic progression and metastasis in a mouse model of breast cancer. Neoplasia. 2011 Jul;13(7):601-10.

doi: 10.1593/neo.11260. PMID: 21750654; PMCID: PMC3132846.

[S12] Akaogi K, Nakajima Y, Ito I, Kawasaki S, Oie SH, Murayama A, Kimura K, Yanagisawa J. KLF4 suppresses estrogen-dependent breast cancer growth by inhibiting the transcriptional activity of ERalpha. Oncogene. 2009 Aug 13;28(32):2894-902. doi: 10.1038/onc.2009.151. Epub 2009 Jun 8. PMID: 19503094.

[S13] Roberts MS, Anstine LJ, Finke VS, Bryson BL, Webb BM, Weber-Bonk KL, Seachrist DD, Majmudar PR, Keri RA. KLF4 defines the efficacy of the epidermal growth factor receptor inhibitor, erlotinib, in triple-negative breast cancer cells by repressing the EGFR gene. Breast Cancer Res. 2020 Jun 18;22(1):66. doi: 10.1186/s13058-020-01305-7. PMID: 32552913; PMCID: PMC7301986.

[S14] Lin CC, Sharma SB, Farrugia MK, McLaughlin SL, Ice RJ, Loskutov YV, Pugacheva EN, Brundage KM, Chen D, Ruppert JM. Kruppel-like factor 4 signals through microRNA-206 to promote tumor initiation and cell survival. Oncogenesis. 2015 Jun 8;4(6):e155. doi: 10.1038/oncsis.2015.8. PMID: 26053033; PMCID: PMC4753526.

[S15] Liu Y, Chen S, Wang S, Soares F, Fischer M, Meng F, Du Z, Lin C, Meyer C, DeCaprio JA, Brown M, Liu XS, He HH. Transcriptional landscape of the human cell cycle. Proc Natl Acad Sci U S A. 2017 Mar 28;114(13):3473-3478. doi: 10.1073/pnas.1617636114. Epub 2017 Mar 13. PMID: 28289232; PMCID: PMC5380023.

[S16] Rowland BD, Bernards R, Peeper DS. The KLF4 tumour suppressor is a transcriptional repressor of p53 that acts as a context-dependent oncogene. Nat Cell Biol. 2005 Nov;7(11):1074-82. doi: 10.1038/ncb1314. PMID: 16244670.

[S17] Yu F, Shi Y, Wang J, Li J, Fan D, Ai W. Deficiency of Kruppel-like factor KLF4 in mammary tumor cells inhibits tumor growth and pulmonary metastasis and is accompanied by compromised recruitment of myeloid-derived suppressor cells. Int J Cancer. 2013 Dec 15;133(12):2872-83. doi: 10.1002/ijc.28302. Epub 2013 Sep 3. PMID: 23737434; PMCID: PMC3796127.

[S18] Meng Z, Liu Y, Wang J, Fan H, Fang H, Li S, Yuan L, Liu C, Peng Y, Zhao W, Wang L, Li J, Feng J. Histone demethylase KDM7A is required for stem cell maintenance and apoptosis inhibition in breast cancer. J Cell Physiol. 2020 Feb;235(2):932-943. doi: 10.1002/jcp.29008. Epub 2019 Jun 24. PMID: 31236965.

[S19] Hu D, Gur M, Zhou Z, Gamper A, Hung MC, Fujita N, Lan L, Bahar I, Wan Y. Interplay between arginine methylation and ubiquitylation regulates KLF4-mediated genome stability and carcinogenesis. Nat Commun. 2015 Sep 30;6:8419. doi: 10.1038/ncomms9419. PMID: 26420673; PMCID: PMC4598737.

[S20] Zou H, Chen H, Zhou Z, Wan Y, Liu Z. ATXN3 promotes breast cancer metastasis by deubiquitinating KLF4. Cancer Lett. 2019 Dec 28;467:19-28. doi: 10.1016/j.canlet.2019.09.012. Epub 2019 Sep 26. PMID: 31563563.

[S21] Singh R, Pochampally R, Watabe K, Lu Z, Mo YY. Exosome-mediated transfer of miR-10b promotes cell invasion in breast cancer. Mol Cancer. 2014 Nov 26;13:256. doi: 10.1186/1476-4598-13-256. PMID: 25428807; PMCID: PMC4258287.

[S22] Shi L, Tang X, Qian M, Liu Z, Meng F, Fu L, Wang Z, Zhu WG, Huang JD, Zhou Z, Liu B. A SIRT1-centered circuitry regulates breast cancer stemness and metastasis. Oncogene. 2018 Dec;37(49):6299-6315. doi: 10.1038/s41388-018-0370-5. Epub 2018 Jul 23. PMID: 30038266; PMCID: PMC6283862.

[S23] Okuda H, Xing F, Pandey PR, Sharma S, Watabe M, Pai SK, Mo YY, Iiizumi-Gairani M, Hirota S, Liu Y, Wu K, Pochampally R, Watabe K. miR-7 suppresses brain metastasis of breast cancer stem-like cells by modulating KLF4. Cancer Res. 2013 Feb 15;73(4):1434-44. doi: 10.1158/0008-5472.CAN-12-2037. Epub 2013 Feb 5.

PMID: 23384942; PMCID: PMC3576138.

[S24] Zhou H, Liu Y, Zhu R, Ding F, Wan Y, Li Y, Liu Z. FBXO32 suppresses breast cancer tumorigenesis through targeting KLF4 to proteasomal degradation. Oncogene. 2017 Jun 8;36(23):3312-3321. doi: 10.1038/onc.2016.479. Epub 2017 Jan 9. PMID: 28068319; PMCID: PMC5926769.

[S25] Pandya AY, Talley LI, Frost AR, Fitzgerald TJ, Trivedi V, Chakravarthy M, Chhieng DC, Grizzle WE, Engler JA, Krontiras H, Bland KI, LoBuglio AF, Lobo-Ruppert SM, Ruppert JM. Nuclear localization of KLF4 is associated with an aggressive phenotype in early-stage breast cancer. Clin Cancer Res. 2004 Apr 15;10(8):2709-19. doi: 10.1158/1078-0432.ccr-03-0484. PMID: 15102675.

[S26] Nagata T, Shimada Y, Sekine S, Moriyama M, Hashimoto I, Matsui K, Okumura T, Hori T, Imura J, Tsukada K. KLF4 and NANOG are prognostic biomarkers for triple-negative breast cancer. Breast Cancer. 2017 Mar;24(2):326-335. doi: 10.1007/s12282-016-0708-1. Epub 2016 Jun 14. PMID: 27300169.

[S27] Wang J, Place RF, Huang V, Wang X, Noonan EJ, Magyar CE, Huang J, Li LC. Prognostic value and function of KLF4 in prostate cancer: RNAa and vector-mediated overexpression identify KLF4 as an inhibitor of tumor cell growth and migration. Cancer Res. 2010 Dec 15;70(24):10182-91. doi: 10.1158/0008-5472.CAN-10-2414. PMID: 21159640; PMCID: PMC3076047.

[S28] Liu YN, Abou-Kheir W, Yin JJ, Fang L, Hynes P, Casey O, Hu D, Wan Y, Seng V, Sheppard-Tillman H, Martin P, Kelly K. Critical and reciprocal regulation of KLF4 and SLUG in transforming growth factor β-initiated prostate cancer epithelial-mesenchymal transition. Mol Cell Biol. 2012 Mar;32(5):941-53. doi: 10.1128/MCB.06306-11. Epub 2011 Dec 27. PMID: 22203039; PMCID: PMC3295188.

[S29] Lee E, Wang J, Yumoto K, Jung Y, Cackowski FC, Decker AM, Li Y, Franceschi RT, Pienta KJ, Taichman RS. DNMT1 Regulates Epithelial-Mesenchymal Transition and Cancer Stem Cells, Which Promotes Prostate Cancer Metastasis. Neoplasia. 2016 Sep;18(9):553-66. doi: 10.1016/j.neo.2016.07.007. PMID: 27659015; PMCID: PMC5031902.

[S30] Lv S, Ji L, Chen B, Liu S, Lei C, Liu X, Qi X, Wang Y, Lai-Han Leung E, Wang H, Zhang L, Yu X, Liu Z, Wei Q, Lu L. Histone methyltransferase KMT2D sustains prostate carcinogenesis and metastasis via epigenetically activating LIFR and KLF4. Oncogene. 2018 Mar;37(10):1354-1368. doi: 10.1038/s41388-017-0026-x. Epub 2017 Dec 22. PMID: 29269867; PMCID: PMC6168472.

[S31] Jiang Z, Zhang Y, Chen X, Wu P, Chen D. Long non-coding RNA LINC00673 silencing inhibits proliferation and drug resistance of prostate cancer cells via decreasing KLF4 promoter methylation. J Cell Mol Med. 2020 Jan;24(2):1878-1892. doi: 10.1111/jcmm.14883. Epub 2019 Dec 27. PMID: 31881124; PMCID: PMC6991650.

[S32] Siu MK, Suau F, Chen WY, Tsai YC, Tsai HY, Yeh HL, Liu YN. KLF4 functions as an activator of the androgen receptor through reciprocal feedback. Oncogenesis. 2016 Dec 19;5(12):e282. doi: 10.1038/oncsis.2016.79. PMID: 27991915; PMCID: PMC5177777.

[S33] Wei LZ, Wang YQ, Chang YL, An N, Wang X, Zhou PJ, Zhu HH, Fang YX, Gao WQ. Imbalance of a KLF4-miR-7 auto-regulatory feedback loop promotes prostate cancer cell growth by impairing microRNA processing. Am J Cancer Res. 2018 Feb 1;8(2):226-244. PMID: 29511594; PMCID: PMC5835691.

[S34] Feng F, Liu H, Chen A, Xia Q, Zhao Y, Jin X, Huang J. miR-148-3p and miR-152-3p synergistically regulate prostate cancer progression via repressing KLF4. J Cell

Biochem. 2019 Oct;120(10):17228-17239. doi: 10.1002/jcb.28984. Epub 2019 May 19. PMID: 31104329.

[S35] Zhang N, Su P, Li X, Xi J, Li X, Xu L. Downregulated Krüppel- like factor 4 expression is associated with the aggressiveness of prostate cancer. Oncol Rep. 2019 Mar;41(3):1789-1796. doi: 10.3892/or.2019.6975. Epub 2019 Jan 22. PMID: 30747213.

[S36] Jiang M, Cheng Y, Wang D, Lu Y, Gu S, Wang C, Huang Y, Li Y. Transcriptional network modulated by the prognostic signature transcription factors and their long noncoding RNA partners in primary prostate cancer. EBioMedicine. 2021 Jan;63:103150. doi: 10.1016/j.ebiom.2020.103150. Epub 2020 Dec 3. PMID: 33279858; PMCID: PMC7718452.

[S37] Xiong X, Schober M, Tassone E, Khodadadi-Jamayran A, Sastre-Perona A, Zhou H, Tsirigos A, Shen S, Chang M, Melamed J, Ossowski L, Wilson EL. KLF4, A Gene Regulating Prostate Stem Cell Homeostasis, Is a Barrier to Malignant Progression and Predictor of Good Prognosis in Prostate Cancer. Cell Rep. 2018 Dec 11;25(11):3006-3020.e7. doi: 10.1016/j.celrep.2018.11.065. PMID: 30540935; PMCID: PMC6405286.

[S38] Le Magnen C, Bubendorf L, Ruiz C, Zlobec I, Bachmann A, Heberer M, Spagnoli GC, Wyler S, Mengus C. Klf4 transcription factor is expressed in the cytoplasm of prostate cancer cells. Eur J Cancer. 2013 Mar;49(4):955-63. doi: 10.1016/j.ejca.2012.09.023. Epub 2012 Oct 22. PMID: 23089465.

[S39] Rong Z, Luo Z, Zhang J, Li T, Zhu Z, Yu Z, Fu Z, Qiu Z, Huang C. GINS complex subunit 4, a prognostic biomarker and reversely mediated by Krüppel-like factor 4, promotes the growth of colorectal cancer. Cancer Sci. 2020 Apr;111(4):1203-1217. doi: 10.1111/cas.14341. Epub 2020 Mar 17. PMID: 32012389; PMCID: PMC7156840.

[S40] Yang VW, Liu Y, Kim J, Shroyer KR, Bialkowska AB. Increased Genetic Instability and Accelerated Progression of Colitis-Associated Colorectal Cancer through Intestinal Epithelium-specific Deletion of Klf4. Mol Cancer Res. 2019 Jan;17(1):165-176. doi: 10.1158/1541-7786.MCR-18-0399. Epub 2018 Aug 14. PMID: 30108164; PMCID: PMC6318049.

[S41] Li D, Peng Z, Tang H, Wei P, Kong X, Yan D, Huang F, Li Q, Le X, Li Q, Xie K. KLF4-mediated negative regulation of IFITM3 expression plays a critical role in colon cancer pathogenesis. Clin Cancer Res. 2011 Jun 1;17(11):3558-68. doi: 10.1158/1078-0432.CCR-10-2729. Epub 2011 Apr 29. PMID: 21531817; PMCID: PMC3107880.

[S42] Xie Y, Zhao J, Liang Y, Chen M, Luo Y, Cui X, Jiang B, Peng L, Wang X. MicroRNA-10b controls the metastasis and proliferation of colorectal cancer cells by regulating Krüppel-like factor 4. Artif Cells Nanomed Biotechnol. 2019 Dec;47(1):1722-1729. doi: 10.1080/21691401.2019.1606006. PMID: 31032663.

[S43] Leng Z, Li Y, Zhou G, Lv X, Ai W, Li J, Hou L. Krüppel-like factor 4 regulates stemness and mesenchymal properties of colorectal cancer stem cells through the TGF-β1/Smad/snail pathway. J Cell Mol Med. 2020 Jan;24(2):1866-1877. doi: 10.1111/jcmm.14882. Epub 2019 Dec 12. PMID: 31830379; PMCID: PMC6991673.

[S44] Tang W, Zhu Y, Gao J, Fu J, Liu C, Liu Y, Song C, Zhu S, Leng Y, Wang G, Chen W, Du P, Huang S, Zhou X, Kang J, Cui L. MicroRNA-29a promotes colorectal cancer metastasis by regulating matrix metalloproteinase 2 and E-cadherin via KLF4. Br J Cancer. 2014 Jan 21;110(2):450-8. doi: 10.1038/bjc.2013.724. Epub 2013 Nov 26. PMID: 24281002; PMCID: PMC3899762.

[S45] Zeng Z, Li Y, Pan Y, Lan X, Song F, Sun J, Zhou K, Liu X, Ren X, Wang F, Hu J, Zhu

X, Yang W, Liao W, Li G, Ding Y, Liang L. Cancer-derived exosomal miR-25-3p promotes pre-metastatic niche formation by inducing vascular permeability and angiogenesis. Nat Commun. 2018 Dec 19;9(1):5395. doi: 10.1038/s41467-018-07810-w. PMID: 30568162; PMCID: PMC6300604.

[S46] Lv H, Zhang Z, Wang Y, Li C, Gong W, Wang X. MicroRNA-92a Promotes Colorectal Cancer Cell Growth and Migration by Inhibiting KLF4. Oncol Res. 2016;23(6):283-90. doi: 10.3727/096504016X14562725373833. PMID: 27131314; PMCID: PMC7838653.

[S47] Chen HY, Lin YM, Chung HC, Lang YD, Lin CJ, Huang J, Wang WC, Lin FM, Chen Z, Huang HD, Shyy JY, Liang JT, Chen RH. miR-103/107 promote metastasis of colorectal cancer by targeting the metastasis suppressors DAPK and KLF4. Cancer Res. 2012 Jul 15;72(14):3631-41. doi: 10.1158/0008-5472.CAN-12-0667. Epub 2012 May 16. Erratum in: Cancer Res. 2017 Dec 1;77(23):6788. PMID: 22593189.

[S48] Ma Y, Wu L, Liu X, Xu Y, Shi W, Liang Y, Yao L, Zheng J, Zhang J. KLF4 inhibits colorectal cancer cell proliferation dependent on NDRG2 signaling. Oncol Rep. 2017 Aug;38(2):975-984. doi: 10.3892/or.2017.5736. Epub 2017 Jun 21. PMID: 28656310.

[S49] Ghaleb AM, Elkarim EA, Bialkowska AB, Yang VW. KLF4 Suppresses Tumor Formation in Genetic and Pharmacological Mouse Models of Colonic Tumorigenesis. Mol Cancer Res. 2016 Apr;14(4):385-96. doi: 10.1158/1541-7786.MCR-15-0410. Epub 2016 Feb 2. PMID: 26839262; PMCID: PMC4834227.

[S50] Agbo KC, Huang JZ, Ghaleb AM, Williams JL, Shroyer KR, Bialkowska AB, Yang VW. Loss of the Krüppel-like factor 4 tumor suppressor is associated with epithelial-mesenchymal transition in colorectal cancer. J Cancer Metastasis Treat. 2019;5:77. doi: 10.20517/2394-4722.2019.35. Epub 2019 Nov 26. PMID: 32566755; PMCID: PMC7304562.

[S51] Guo K, Cui J, Quan M, Xie D, Jia Z, Wei D, Wang L, Gao Y, Ma Q, Xie K. The Novel KLF4/MSI2 Signaling Pathway Regulates Growth and Metastasis of Pancreatic Cancer. Clin Cancer Res. 2017 Feb 1;23(3):687-696. doi: 10.1158/1078-0432.CCR-16-1064. Epub 2016 Jul 22. PMID: 27449499; PMCID: PMC5253336.

[S52] Yan Y, Li Z, Kong X, Jia Z, Zuo X, Gagea M, Huang S, Wei D, Xie K. KLF4-Mediated Suppression of CD44 Signaling Negatively Impacts Pancreatic Cancer Stemness and Metastasis. Cancer Res. 2016 Apr 15;76(8):2419-31. doi: 10.1158/0008-5472.CAN-15-1691. Epub 2016 Feb 15. PMID: 26880805; PMCID: PMC4876033.

[S53] Zhu Z, Yu Z, Wang J, Zhou L, Zhang J, Yao B, Dou J, Qiu Z, Huang C. Krüppel-Like Factor 4 Inhibits Pancreatic Cancer Epithelial-to-Mesenchymal Transition and Metastasis by Down-Regulating Caveolin-1 Expression. Cell Physiol Biochem. 2018;46(1):238-252. doi: 10.1159/000488426. Epub 2018 Mar 21. PMID: 29587259.

[S54] Wei D, Kanai M, Jia Z, Le X, Xie K. Kruppel-like factor 4 induces p27Kip1 expression in and suppresses the growth and metastasis of human pancreatic cancer cells. Cancer Res. 2008 Jun 15;68(12):4631-9. doi: 10.1158/0008-5472.CAN-07-5953. PMID: 18559508; PMCID: PMC2481517.

[S55] Xie VK, Li Z, Yan Y, Jia Z, Zuo X, Ju Z, Wang J, Du J, Xie D, Xie K, Wei D. DNA-Methyltransferase 1 Induces Dedifferentiation of Pancreatic Cancer Cells through Silencing of Krüppel-Like Factor 4 Expression. Clin Cancer Res. 2017 Sep 15;23(18):5585-5597. doi: 10.1158/1078-0432.CCR-17-0387. Epub 2017 Jun 28. PMID: 28659310; PMCID: PMC5600846.

[S56] Ganguly K, Krishn SR, Rachagani S, Jahan R, Shah A, Nallasamy P, Rauth S, Atri P,

Cox JL, Pothuraju R, Smith LM, Ayala S, Evans C, Ponnusamy MP, Kumar S, Kaur S, Batra SK. Secretory Mucin 5AC Promotes Neoplastic Progression by Augmenting KLF4-Mediated Pancreatic Cancer Cell Stemness. Cancer Res. 2021 Jan 1;81(1):91-102. doi: 10.1158/0008-5472.CAN-20-1293. Epub 2020 Oct 30. PMID: 33127746; PMCID: PMC7990052.

[S57] Min KW, Zhang X, Imchen T, Baek SJ. A peroxisome proliferator-activated receptor ligand MCC-555 imparts anti-proliferative response in pancreatic cancer cells by PPARgamma-independent up-regulation of KLF4. Toxicol Appl Pharmacol. 2012 Sep 1;263(2):225-32. doi: 10.1016/j.taap.2012.06.014. Epub 2012 Jun 30. PMID: 22750490; PMCID: PMC3443873.

[S58] Lowenfels AB, Maisonneuve P, Cavallini G, Ammann RW, Lankisch PG, Andersen JR, Dimagno EP, Andrén-Sandberg A, Domellöf L. Pancreatitis and the risk of pancreatic cancer. International Pancreatitis Study Group. N Engl J Med. 1993 May 20;328(20):1433-7. doi: 10.1056/NEJM199305203282001. PMID: 8479461.

[S59] Guerra C, Schuhmacher AJ, Cañamero M, Grippo PJ, Verdaguer L, Pérez-Gallego L, Dubus P, Sandgren EP, Barbacid M. Chronic pancreatitis is essential for induction of pancreatic ductal adenocarcinoma by K-Ras oncogenes in adult mice. Cancer Cell. 2007 Mar;11(3):291-302. doi: 10.1016/j.ccr.2007.01.012. PMID: 17349585.

[S60] Morris JP 4th, Cano DA, Sekine S, Wang SC, Hebrok M. Beta-catenin blocks Kras-dependent reprogramming of acini into pancreatic cancer precursor lesions in mice. J Clin Invest. 2010 Feb;120(2):508-20. doi: 10.1172/JCI40045. Epub 2010 Jan 11. PMID: 20071774; PMCID: PMC2810083.

[S61] Yang Z, Li D, Liu Z, Miao X, Yang L, Zou Q, Yuan Y. BIRC7 and KLF4 expression in benign and malignant lesions of pancreas and their clinicopathological significance. Cancer Biomark. 2016;17(4):437-444. doi: 10.3233/CBM-160660. PMID: 27802195.

[S62] Sun H, Peng Z, Tang H, Xie D, Jia Z, Zhong L, Zhao S, Ma Z, Gao Y, Zeng L, Luo R, Xie K. Loss of KLF4 and consequential downregulation of Smad7 exacerbate oncogenic TGF-β signaling in and promote progression of hepatocellular carcinoma. Oncogene. 2017 May 25;36(21):2957-2968. doi: 10.1038/onc.2016.447. Epub 2017 Feb 13. PMID: 28192402; PMCID: PMC5444978.

[S63] Li Q, Gao Y, Jia Z, Mishra L, Guo K, Li Z, Le X, Wei D, Huang S, Xie K. Dysregulated Krüppel-like factor 4 and vitamin D receptor signaling contribute to progression of hepatocellular carcinoma. Gastroenterology. 2012 Sep;143(3):799-810.e2. doi: 10.1053/j.gastro.2012.05.043. Epub 2012 Jun 4. PMID: 22677193; PMCID: PMC3653768.

[S64] Sung MT, Hsu HT, Lee CC, Lee HC, Kuo YJ, Hua K, Hsia CY, Chi CW. Krüppel-like factor 4 modulates the migration and invasion of hepatoma cells by suppressing TIMP-1 and TIMP-2. Oncol Rep. 2015 Jul;34(1):439-46. doi: 10.3892/or.2015.3964. Epub 2015 May 8. PMID: 25954999.

[S65] Tian C, Yao S, Liu L, Ding Y, Ye Q, Dong X, Gao Y, Yang N, Li Q. Klf4 inhibits tumor growth and metastasis by targeting microRNA-31 in human hepatocellular carcinoma. Int J Mol Med. 2017 Jan;39(1):47-56. doi: 10.3892/ijmm.2016.2812. Epub 2016 Nov 24. PMID: 27909734; PMCID: PMC5179175.

[S66] Yang X, Zhang D, Liu S, Li X, Hu W, Han C. KLF4 suppresses the migration of hepatocellular carcinoma by transcriptionally upregulating monoglyceride lipase. Am J Cancer Res. 2018 Jun 1;8(6):1019-1029. PMID: 30034939; PMCID: PMC6048399.

[S67] Li Y, Yu S, Li L, Chen J, Quan M, Li Q, Gao Y. KLF4-mediated upregulation of CD9 and CD81 suppresses hepatocellular carcinoma development via JNK signaling. Cell Death Dis. 2020 Apr 29;11(4):299. doi: 10.1038/s41419-020-2479-z. PMID:

32350244; PMCID: PMC7190708.

[S68] Li L, Yu S, Wu Q, Dou N, Li Y, Gao Y. KLF4-Mediated CDH3 Upregulation Suppresses Human Hepatoma Cell Growth and Migration via GSK-3β Signaling. Int J Biol Sci. 2019 Mar 10;15(5):953-961. doi: 10.7150/ijbs.30857. PMID: 31182916; PMCID: PMC6535787.

[S69] Chen X, Ding X, Wu Q, Qi J, Zhu M, Miao C. Monomethyltransferase SET8 facilitates hepatocellular carcinoma growth by enhancing aerobic glycolysis. Cell Death Dis. 2019 Apr 5;10(4):312. doi: 10.1038/s41419-019-1541-1. PMID: 30952833; PMCID: PMC6450876.

[S70] Xue Y, Jia X, Li C, Zhang K, Li L, Wu J, Yuan J, Li Q. DDX17 promotes hepatocellular carcinoma progression via inhibiting Klf4 transcriptional activity. Cell Death Dis. 2019 Oct 25;10(11):814. doi: 10.1038/s41419-019-2044-9. PMID: 31653828; PMCID: PMC6814716.

[S71] Dong X, Wang F, Xue Y, Lin Z, Song W, Yang N, Li Q. MicroRNA- 9- 5p downregulates Klf4 and influences the progression of hepatocellular carcinoma via the AKT signaling pathway. Int J Mol Med. 2019 Mar;43(3):1417-1429. doi: 10.3892/ijmm.2019.4062. Epub 2019 Jan 14. PMID: 30664155; PMCID: PMC6365078.

[S72] He H, Wu Z, Li S, Chen K, Wang D, Zou H, Chen H, Li Y, Liu Z, Qu C. TRAF7 enhances ubiquitin-degradation of KLF4 to promote hepatocellular carcinoma progression. Cancer Lett. 2020 Jan 28;469:380-389. doi: 10.1016/j.canlet.2019.11.012. Epub 2019 Nov 12. PMID: 31730901.

[S73] Xue M, Zhou C, Zheng Y, Zhang Z, Wang S, Fu Y, Atyah M, Xue X, Zhu L, Dong Q, Jia H, Ren N, Hu R. The association between KLF4 as a tumor suppressor and the prognosis of hepatocellular carcinoma after curative resection. Aging (Albany NY). 2020 Aug 5;12(15):15566-15580. doi: 10.18632/aging.103592. Epub 2020 Aug 5. PMID: 32756012; PMCID: PMC7467357.

[S74] Chen Z, Wang Y, Liu W, Zhao G, Lee S, Balogh A, Zou Y, Guo Y, Zhang Z, Gu W, Li C, Tigyi G, Yue J. Doxycycline inducible Krüppel-like factor 4 lentiviral vector mediates mesenchymal to epithelial transition in ovarian cancer cells. PLoS One. 2014 Aug 19;9(8):e105331. doi: 10.1371/journal.pone.0105331. PMID: 25137052; PMCID: PMC4138168.

[S75] Yoon O, Roh J. Downregulation of KLF4 and the Bcl-2/Bax ratio in advanced epithelial ovarian cancer. Oncol Lett. 2012 Nov;4(5):1033-1036. doi: 10.3892/ol.2012.834. Epub 2012 Jul 30. PMID: 23162646; PMCID: PMC3499599.

[S76] Zhang C, Liu J, Zhang Y, Luo C, Zhu T, Zhang R, Yao R. LINC01210 accelerates proliferation, invasion and migration in ovarian cancer through epigenetically downregulating KLF4. Biomed Pharmacother. 2019 Nov;119:109431. doi: 10.1016/j.biopha.2019.109431. Epub 2019 Sep 9. PMID: 31514068.

[S77] Zhang L, Zhou Q, Qiu Q, Hou L, Wu M, Li J, Li X, Lu B, Cheng X, Liu P, Lu W, Lu Y. CircPLEKHM3 acts as a tumor suppressor through regulation of the miR-9/BRCA1/DNAJB6/KLF4/AKT1 axis in ovarian cancer. Mol Cancer. 2019 Oct 17;18(1):144. doi: 10.1186/s12943-019-1080-5. PMID: 31623606; PMCID: PMC6796346.

[S78] Zhang X, Chen J, Sun L, Xu Y. SIRT1 deacetylates KLF4 to activate Claudin-5 transcription in ovarian cancer cells. J Cell Biochem. 2018 Feb;119(2):2418-2426. doi: 10.1002/jcb.26404. Epub 2017 Oct 17. PMID: 28888043.

[S79] Zhang G, Zhu H, Wang Y, Yang S, Liu M, Zhang W, Quan L, Bai J, Liu Z, Xu N. Krüppel-like factor 4 represses transcription of the survivin gene in esophageal cancer cell lines. Biol Chem. 2009 May-Jun;390(5-6):463-9. doi:

10.1515/BC.2009.060. PMID: 19361279.

[S80] He H, Li S, Hong Y, Zou H, Chen H, Ding F, Wan Y, Liu Z. Krüppel-like Factor 4 Promotes Esophageal Squamous Cell Carcinoma Differentiation by Up-regulating Keratin 13 Expression. J Biol Chem. 2015 May 22;290(21):13567-77. doi: 10.1074/jbc.M114.629717. Epub 2015 Apr 7. PMID: 25851906; PMCID: PMC4505602.

[S81] Tian Y, Luo A, Cai Y, Su Q, Ding F, Chen H, Liu Z. MicroRNA-10b promotes migration and invasion through KLF4 in human esophageal cancer cell lines. J Biol Chem. 2010 Mar 12;285(11):7986-94. doi: 10.1074/jbc.M109.062877. Epub 2010 Jan 14. PMID: 20075075; PMCID: PMC2832949.

[S82] Wang W, Fu S, Lin X, Zheng J, Pu J, Gu Y, Deng W, Liu Y, He Z, Liang W, Wang C. miR-92b-3p Functions As A Key Gene In Esophageal Squamous Cell Cancer As Determined By Co-Expression Analysis. Onco Targets Ther. 2019 Oct 14;12:8339-8353. doi: 10.2147/OTT.S220823. PMID: 31686859; PMCID: PMC6799829.

[S83] Huang H, Wei L, Qin T, Yang N, Li Z, Xu Z. Circular RNA ciRS-7 triggers the migration and invasion of esophageal squamous cell carcinoma via miR-7/KLF4 and NF-κB signals. Cancer Biol Ther. 2019;20(1):73-80. doi: 10.1080/15384047.2018.1507254. Epub 2018 Sep 12. PMID: 30207835; PMCID: PMC6343722.

[S84] Yang Y, Katz JP. KLF4 is downregulated but not mutated during human esophageal squamous cell carcinogenesis and has tumor stage-specific functions. Cancer Biol Ther. 2016 Apr 2;17(4):422-9. doi: 10.1080/15384047.2016.1156260. Epub 2016 Mar 2. PMID: 26934576; PMCID: PMC4910921.

[S85] Yang Y, Goldstein BG, Chao HH, Katz JP. KLF4 and KLF5 regulate proliferation, apoptosis and invasion in esophageal cancer cells. Cancer Biol Ther. 2005 Nov;4(11):1216-21. doi: 10.4161/cbt.4.11.2090. Epub 2005 Nov 11. PMID: 16357509.

[S86] Shi W, Song J, Gao Z, Liu X, Wang W. Downregulation of miR-7-5p Inhibits the Tumorigenesis of Esophagus Cancer via Targeting KLF4. Onco Targets Ther. 2020 Sep 24;13:9443-9453. doi: 10.2147/OTT.S251508. PMID: 33061430; PMCID: PMC7522318.

[S87] Tseng WC, Chuang CW, Yang MH, Pan CC, Tarng DC. Krüppel-like factor 4 is a novel prognostic predictor for urothelial carcinoma of bladder and it regulates TWIST1-mediated epithelial-mesenchymal transition. Urol Oncol. 2016 Nov;34(11):485.e15-485.e24. doi: 10.1016/j.urolonc.2016.07.002. Epub 2016 Aug 9. PMID: 27519276.

[S88] Li H, Wang J, Xiao W, Xia D, Lang B, Wang T, Guo X, Hu Z, Ye Z, Xu H. Epigenetic inactivation of KLF4 is associated with urothelial cancer progression and early recurrence. J Urol. 2014 Feb;191(2):493-501. doi: 10.1016/j.juro.2013.08.087. Epub 2013 Sep 7. PMID: 24018236.

[S89] Xiao H, Li H, Yu G, Xiao W, Hu J, Tang K, Zeng J, He W, Zeng G, Ye Z, Xu H. MicroRNA-10b promotes migration and invasion through KLF4 and HOXD10 in human bladder cancer. Oncol Rep. 2014 Apr;31(4):1832-8. doi: 10.3892/or.2014.3048. Epub 2014 Feb 24. Erratum in: Oncol Rep. 2022 Jul;48(1): PMID: 24573354.

[S90] Xie H, Li J, Ying Y, Yan H, Jin K, Ma X, He L, Xu X, Liu B, Wang X, Zheng X, Xie L. METTL3/YTHDF2 m6 A axis promotes tumorigenesis by degrading SETD7 and KLF4 mRNAs in bladder cancer. J Cell Mol Med. 2020 Apr;24(7):4092-4104. doi: 10.1111/jcmm.15063. Epub 2020 Mar 3. PMID: 32126149; PMCID: PMC7171394.

[S91] Ai X, Jia Z, Liu S, Wang J, Zhang X. Notch-1 regulates proliferation and differentiation of human bladder cancer cell lines by inhibiting expression of Krüppel-like factor 4. Oncol Rep. 2014 Oct;32(4):1459-64. doi: 10.3892/or.2014.3350. Epub 2014 Jul 23. PMID: 25109409.

[S92] Jia ZM, Ai X, Teng JF, Wang YP, Wang BJ, Zhang X. p21 and CK2 interaction-mediated HDAC2 phosphorylation modulates KLF4 acetylation to regulate bladder cancer cell proliferation. Tumour Biol. 2016 Jun;37(6):8293-304. doi: 10.1007/s13277-015-4618-1. Epub 2016 Jan 4. PMID: 26729194.

[S93] Ohnishi S, Ohnami S, Laub F, Aoki K, Suzuki K, Kanai Y, Haga K, Asaka M, Ramirez F, Yoshida T. Downregulation and growth inhibitory effect of epithelial-type Krüppel-like transcription factor KLF4, but not KLF5, in bladder cancer. Biochem Biophys Res Commun. 2003 Aug 22;308(2):251-6. doi: 10.1016/s0006-291x(03)01356-1. PMID: 12901861.

[S94] von Spreckelsen N, Waldt N, Poetschke R, Kesseler C, Dohmen H, Jiao HK, Nemeth A, Schob S, Scherlach C, Sandalcioglu IE, Deckert M, Angenstein F, Krischek B, Stavrinou P, Timmer M, Remke M, Kirches E, Goldbrunner R, Chiocca EA, Huettelmaier S, Acker T, Mawrin C. KLF4K409Q-mutated meningiomas show enhanced hypoxia signaling and respond to mTORC1 inhibitor treatment. Acta Neuropathol Commun. 2020 Apr 3;8(1):41. doi: 10.1186/s40478-020-00912-x. PMID: 32245394; PMCID: PMC7118946.

[S95] Mohan N, Ai W, Chakrabarti M, Banik NL, Ray SK. KLF4 overexpression and apigenin treatment down regulated anti-apoptotic Bcl-2 proteins and matrix metalloproteinases to control growth of human malignant neuroblastoma SK-N-DZ and IMR-32 cells. Mol Oncol. 2013 Jun;7(3):464-74. doi: 10.1016/j.molonc.2012.12.002. Epub 2012 Dec 20. PMID: 23317647; PMCID: PMC3634908.

[S96] Nakahara Y, Northcott PA, Li M, Kongkham PN, Smith C, Yan H, Croul S, Ra YS, Eberhart C, Huang A, Bigner D, Grajkowska W, Van Meter T, Rutka JT, Taylor MD. Genetic and epigenetic inactivation of Kruppel-like factor 4 in medulloblastoma. Neoplasia. 2010 Jan;12(1):20-7. doi: 10.1593/neo.91122. PMID: 20072650; PMCID: PMC2805880.

[S97] Tang H, Zhu H, Wang X, Hua L, Li J, Xie Q, Chen X, Zhang T, Gong Y. KLF4 is a tumor suppressor in anaplastic meningioma stem-like cells and human meningiomas. J Mol Cell Biol. 2017 Aug 1;9(4):315-324. doi: 10.1093/jmcb/mjx023. PMID: 28651379.

[S98] Wang S, Shi X, Wei S, Ma D, Oyinlade O, Lv SQ, Ying M, Zhang YA, Claypool SM, Watkins P, Xia S. Krüppel-like factor 4 (KLF4) induces mitochondrial fusion and increases spare respiratory capacity of human glioblastoma cells. J Biol Chem. 2018 Apr 27;293(17):6544-6555. doi: 10.1074/jbc.RA117.001323. Epub 2018 Mar 5. PMID: 29507094; PMCID: PMC5925822.

[S99] Clark VE, Erson-Omay EZ, Serin A, Yin J, Cotney J, Ozduman K, Avşar T, Li J, Murray PB, Henegariu O, Yilmaz S, Günel JM, Carrión-Grant G, Yilmaz B, Grady C, Tanrikulu B, Bakircioğlu M, Kaymakçalan H, Caglayan AO, Sencar L, Ceyhun E, Atik AF, Bayri Y, Bai H, Kolb LE, Hebert RM, Omay SB, Mishra-Gorur K, Choi M, Overton JD, Holland EC, Mane S, State MW, Bilgüvar K, Baehring JM, Gutin PH, Piepmeier JM, Vortmeyer A, Brennan CW, Pamir MN, Kiliç T, Lifton RP, Noonan JP, Yasuno K, Günel M. Genomic analysis of non-NF2 meningiomas reveals mutations in TRAF7, KLF4, AKT1, and SMO. Science. 2013 Mar 1;339(6123):1077-80. doi: 10.1126/science.1233009. Epub 2013 Jan 24. PMID: 23348505; PMCID: PMC4808587.
